# Does Working in a COVID-19 Receiving Health Facility Influence Seroprevalence to SARS-CoV-2?

**DOI:** 10.7759/cureus.11389

**Published:** 2020-11-09

**Authors:** Mohammad Noor, Mohsina Haq, Najib Ul Haq, Said Amin, Fawad Rahim, Sher Bahadur, Raza Ullah, Muhammad Asif Khan, Afsheen Mahmood, Huma Gul

**Affiliations:** 1 Internal Medicine, Hayatabad Medical Complex, Peshawar, PAK; 2 Internal Medicine, Khyber Girls Medical College, Peshawar, PAK; 3 Microbiology, Peshawar Medical College, Peshawar, PAK; 4 Medicine, Peshawar Medical College, Peshawar, PAK; 5 Epidemiology and Public Health, Khyber Institute of Child Health, Peshawar, PAK; 6 Critical Care Medicine, Hayatabad Medical Complex, Peshawar, PAK

**Keywords:** covid-19, sars-cov-2, seroprevalence, health care workers

## Abstract

Objective

In this study, we aimed at comparing the seroprevalence of severe acute respiratory syndrome coronavirus 2 (SARS-CoV-2) antibodies in healthcare workers (HCWs) in coronavirus disease 2019 (COVID-19) receiving and non-COVID-19 receiving hospitals in Peshawar, Pakistan.

Methods

This cross-sectional analytical study was conducted in a COVID-19 receiving hospital (hospital ‘A’) and a non-COVID-19 receiving hospital (hospital ‘B’). Using stratified random sampling, 1,011 HCWs (439 from hospital ‘A’ and 572 from hospital ‘B’) were recruited to participate in the study. Immunoglobulin G/immunoglobulin M (IgG/IgM) antibodies were checked using Elecsys® (Roche, Basel, Switzerland) Anti-SARS-CoV-2 immunoassay. The chi-squared test was used to compare frequencies, and the binary logistic regression model was used to predict the association between study variables' seropositivity to SARS-CoV-2. A p-value of <0.05 was considered statistically significant.

Results

The overall seroprevalence to SARS-CoV-2 antibodies in the two hospitals was 30.76%. It was 28.2% in hospital ‘A’ and 32.7% in hospital ‘B’ (p=0.129). The seroprevalence in HCWs having direct contact with COVID-19 patients was higher (33.1%) in non-COVID-19 receiving hospital versus 23.8% in COVID-19 receiving hospital (p=0.034). Seroprevalence was highest among administrative staff (44.0%), followed by nurses (30.8%), residents (19.8%), and consultants (17.8%) (p=0.001). As compared to consultants, the administrative and nursing staff were 3.398 and 3.116 times more likely to have positive antibodies, respectively. There were no significant differences in the seroprevalence between the respective categories of staff of the two hospitals.

Conclusions

The non-COVID-19 receiving hospital had a higher proportion of seropositive HCWs than the COVID-19 receiving hospital. The HCWs in the non-COVID-19 receiving hospital who had direct contact with patients had significantly higher seroprevalence. Seroprevalence was highest for administrative staff followed by nursing staff, residents, and consultants. Regardless of the COVID-19 status of the healthcare facility, all HCWs shall be trained on, and consistently follow, the proper protocols for donning and doffing of personal protective equipment (PPE).

## Introduction

Coronavirus disease 19 (COVID-19) originated in the city of Wuhan, China [1], and was declared as a global pandemic by the World Health Organization (WHO) on March 11, 2020 [2]. COVID-19 has not only adversely affected the global economy but has also resulted in substantial morbidity and mortality among humans. The World Bank has predicted that the world economy will decline by 5.2% in 2020, resulting in the deepest economic recession since World War II [3]. As of September 6, 2020, severe acute respiratory syndrome coronavirus 2 (SARS-CoV-2), the virus that causes COVID-19, has infected 26,763,217 people and has resulted in the death of 876,616 people worldwide [4]. Pakistan reported its first COVID-19 case on February 26, 2020, and as of September 6, 2020, there have been 274,287 reported cases of COVID-19 in the country; and the death tally in Pakistan currently stands at 6,342 [5]. The seroprevalence from a community survey in Pakistan was found to be 0.2% (95% CI: 0-0.7) in low-transmission areas and 0.4% (95% CI: 0-1.3) in high-transmission areas in the early phase, and 8.7% (95% CI: 5.1-13.1) in low-transmission areas and 15.1% (95% CI: 9.4-21.7) in high-transmission areas in the late phase of the pandemic [6]. A countrywide survey revealed that the seroprevalence was 11% among the general population in Pakistan [7].

Healthcare workers (HCWs) are at a higher risk of contracting SARS-CoV-2 infection due to their close contact with the COVID-19 confirmed/pre-symptomatic/asymptomatic patients/colleagues in addition to other risk factors [8]. In China, out of 77,262 cases of COVID-19, 3,387 (4.4%) were HCWs as of February 24, 2020 [9]. In South Korea, a total of 121 HCWs got infected with an infection rate of 4.42 cases per 1,000 people compared to 2.72 cases in the general population [10]. In a tertiary care hospital in Germany, the seroprevalence of SARS-CoV-2 antibodies among the HCWs was 1.6% [11]. Studies from Spain, Italy, Greece, and the US have reported high SARS-CoV-2 infection rates in HCWs, ranging from 5 to 44% [12-15]. Research conducted by Papoutsi et al. revealed that over 67,569 HCWs have been infected by SARS-CoV-2 worldwide [16]. Surprisingly, Asian countries have reported lower infection rates among HCWs, which may be due to different testing and reporting policies compared to the developed countries in the west [16]. Preventing infections among HCWs is crucial for reducing morbidity and potential mortality, not only for maintaining the dynamics of the health system and human resource capacity during the pandemic but also for reducing secondary transmission to patients, colleagues, and the general public [17]. It has been reported from Hongkong that the seroprevalence to SARS-CoV-1 was higher in HCWs working in SARS medical wards compared to those working in general medical wards [18].

There is a general perception that HCWs working in a COVID-19-receiving healthcare facility are at a higher risk of contracting COVID-19 as compared to those working in a non-COVID-19-receiving facility. There is a paucity of research on the HCWs from developing countries of South Asia including Pakistan. This study aimed to compare the seroprevalence of SARS-CoV-2 antibodies among the HCWs in a COVID-19-receiving hospital (hospital ‘A’) and a non-COVID-19-receiving hospital (hospital ‘B’) in the city of Peshawar, Pakistan.

## Materials and methods

Operational definitions

For the purpose of our study, a COVID-19 receiving hospital was defined as a hospital offering facilities of screening, diagnosis, in-patient care, and intensive care services for suspected and confirmed cases of COVID-19. A non-COVID-19 receiving hospital was defined as a hospital with only triage and referral services for suspected and confirmed COVID-19 cases.

The HCW was defined as any regular employee of the two hospitals irrespective of their involvement in the clinical management of COVID-19 patients. The administrative staff was defined as all regular employees of the hospitals who were doing clerical and administrative work in offices. Direct contact of HCW was defined as the interaction of the HCW with suspected or confirmed cases of COVID-19.

Study design

This was a cross-sectional analytical study carried out in two tertiary care hospitals in Peshawar, Pakistan. Hayatabad Medical Complex, Peshawar (hospital ‘A’) is one of the major COVID-19 receiving hospitals in Pakistan. It is a 1,200-bedded tertiary care facility employing 2,800 HCWs. We chose an affiliated teaching hospital of Peshawar Medical College, Peshawar as hospital ‘B’. It is a non-COVID-19 receiving hospital offering only screening and referral facilities for suspected COVID-19 patients. It is a 600-bedded hospital with 1,050 HCWs. After triage, suspected COVID-19 patients from hospital ‘B’ were referred to COVID-19 receiving hospitals of Peshawar city. Both hospitals mostly cater to populations of the same ethnic background. The study was approved by the Institutional Review Board and the Institutional Ethical Committee of the respective hospitals. All regular HCWs of hospital ‘A’ and hospital ‘B’, regardless of their age and gender, were considered eligible for the study. HCWs with conditions affecting immunity, such as HIV infection or the use of chemotherapeutic agents and steroids, were excluded from the study.

Sample size and sampling technique

A sample size of 1,051 was arrived at by using the WHO sample size calculator, assuming a prevalence rate of 33.8% [19], a confidence level of 95%, a margin of error of 3%, and an addition of 10% to compensate for the loss to follow-up. Using the non-proportionate stratified random sampling, 1,051 HCWs (469 from hospital ‘A’ and 582 from hospital ‘B’) were approached for participation in the study.

Data collection procedure

Data were collected from July 13 to 25, 2020. Informed written consent was obtained from every participant. A structured proforma was provided to all participants for recording demographic parameters and other risk factors for contracting the virus. A blood sample of 5 ml was obtained from each participant, which was kept in lithium heparin bottles. The sample was immediately centrifuged, stored at a temperature of -80 °C, and analyzed using Elecsys® (Roche, Basel, Switzerland) Anti-SARS-CoV-2 immunoassay (sensitivity of 100% and specificity of 99.8%) [20] for the quantitative detection of Immunoglobulin G/immunoglobulin M (IgG/IgM) antibodies against SARS-CoV-2. An independent evaluation by the UK Public Health has estimated its sensitivity and specificity to be 87% and 100%, respectively [21]. For quality assurance, positive and negative controlled tests were carried out. The cut-off for significant antibodies level was taken as 1 or more as per manufacturer instruction. The participants were informed in person about the results of the antibodies' levels.

Statistical analysis

The data were analyzed using SPSS Statistics version 21 (IBM, Armonk, NY). The chi-squared test was used to determine the statistical significance of the differences between the groups. The binary logistic regression model was used to predict the association between study variables and the frequency of having positive antibodies to SARS-CoV-2.

## Results

Out of the 1,051 participants, 1,011 completed the sampling protocol, indicating a response rate of 98.2%. The mean age and gender-wise distribution of the study population of both hospitals were almost identical. The demographic characteristics of the study participants are outlined in Table 1.

**Table 1:** Demographic characteristics of the study population (n=1,101) COVID-19: coronavirus disease 2019; SD: standard deviation

Characteristics	COVID-19 receiving hospital (n=439 participants)	Non-COVID-19 receiving hospital (n=572 participants)
Mean age in years (mean ± SD)	33.25 ± 8.71	33.94 ± 11.77
Age groups		
Minimum-29 years, n (%)	184 (41.9%)	272 (47.6%)
30-39 years, n (%)	159 (36.2%)	150 (26.2%)
40-49 years, n (%)	61 (13.9%)	77 (13.5%)
50-59 years, n (%)	34 (7.7%)	37 (6.5%)
60-69 years, n (%)	1 (0.2%)	33 (5.8%)
70-79 years, n (%)	0 (0%)	3 (0.5%)
Gender		
Male, n (%)	285 (64.9%)	403 (70.5%)
Female, n (%)	154 (35.1%)	169 (29.5%)
Staff category		
Consultants, n (%)	68 (15.5%)	89 (15.6%)
Residents, n (%)	166 (37.8%)	137 (24.0%)
Nursing staff, n (%)	183 (41.7%)	277 (48.4%)
Administrative staff, n (%)	22 (5.0%)	69 (12.1%)

The overall seroprevalence of SARS-CoV-2 antibodies in HCWs of both hospitals was 30.76%. It was 28.8% in hospital ‘A’ and 32.7% in hospital ‘B’, with a p-value of 0.11. Among HCWs in the age group of less than 50 years, seroprevalence for SARS-CoV-2 antibodies was 28.0% in hospital ‘A’ and 33.7% in hospital ‘B’ (p=0.066). Similarly, no statistical difference (p=0.558) was observed among the age group of more than 50 years. The gender-wise difference in the seroprevalence among HCWs of both hospitals was not significant (p: >0.05). The only significant difference was observed among HCWs who had direct contact with COVID-19 patients. The seroprevalence was high (33.1%) in non-COVID-19 receiving hospital as compared to 23.8% in COVID-19 receiving hospital (p=0.034). An insignificant difference (p: >0.05) was observed in the seroprevalence of SARS-CoV-2 antibodies among different categories of HCWs in the two hospitals. However, the overall seroprevalence was highest for administrative staff (44.0%), followed by nursing staff (39.8%), residents (19.8%), and consultants (17.8%) (p=0.001) (Table 2).

**Table 2:** Comparison of levels of antibodies among HCWs of hospital ‘A’ and hospital ‘B’ COVID-19: coronavirus disease 2019; HCWs: healthcare workers

Parameters	Hospital ‘A’ (COVID-19 receiving)		Hospital ‘B’ (non-COVID-19 receiving)		P-value
	Levels of antibodies		Levels of antibodies		
	Significant level (>1 = positive), n (%)	Insignificant level (<1 = negative), n (%)	Significant level (>1 = positive), n (%)	Insignificant level (<1 = negative), n (%)	
All HCWs	124 (28.2%)	315 (71.8%)	187 (32.7%)	385 (67.3%)	0.129
Age groups					
Age up to 50 years	113 (28.0%)	291 (72.0%)	168 (33.7%)	331 (67.3%)	0.066
Age over 50 years	11 (31.4%)	24 (68.6%)	19 (26.0%)	54 (74.0%)	0.558
Gender					
Male	83 (29.1%)	202 (70.9%)	136 (33.7%)	267 (66.3%)	0.200
Female	41 (26.6%)	113 (73.4%)	51 (30.2%)	118 (69.8%)	0.480
Contact status					
Direct contact with patients	73 (23.8%)	234 (76.2%)	50 (33.1%)	101 (66.9%)	0.034
No direct contact with patients	51 (38.6%)	81 (61.4%)	137 (32.5%)	284 (67.5%)	0.197
Categories of hospital staff					
Consultants	15 (22.1%)	53 (77.9%)	13 (14.6%)	76 (85.4%)	0.227
Residents and medical officers	27 (16.3%)	139 (83.7%)	33 (24.1%)	104 (75.9%)	0.089
Nursing and allied	70 (38.3%)	113 (61.7%)	113 (40.8%)	164 (59.2%)	0.585
Administrative staff	12 (54.5%)	10 (45.5%)	28 (40.6%)	41 (59.4%)	0.250

The regression analysis showed that the HCWs of both hospitals who had no direct contact with COVID-19 patients was 1.186 (95% CI: 0.866-1.622) times more likely to have positive antibodies. As compared to consultants, the residents, nurses, and administrative staff were 1.190 (95% CI: 0.701-2.019), 3.116 (95% CI: 1.939-5.006), and 3.398 (95% CI: 1.863-6.199) times more likely to have positive antibodies. Overall, the odds of having positive antibodies to SARS-CoV-2 were less than 1 among females, those over 50 years of age, and those working in the non-COVID-19 receiving hospital (Table 3).

**Table 3:** Logistic regression analysis of SARS-CoV-2 antibodies for gender, age, contact status, and job categories COVID-19: coronavirus disease 2019; OR: odds ratio; SARS-CoV-2: severe acute respiratory syndrome coronavirus 2

Relative variables	OR	95% confidence interval
Female/male	0.811	0.596-1.104
Non-COVID-19 receiving (hospital 'B')/COVID-19 receiving (hospital 'A')	0.991	0.725-1.353
More than 50 years/less than 50 years of age	0.939	0.576-1.532
No direct contact/direct contact	1.186	0.866-1.622
Residents/consultants	1.190	0.701-2.019
Nursing staff/consultants	3.116	1.939-5.006
Administrative staff/consultants	3.398	1.863-6.199

## Discussion

Despite using personal protective equipment (PPE), HCWs have a high risk of contracting SARS-CoV-2 in comparison to the general public as reported from different regions of the world [17]. It not only causes human resource depletion during the outbreak but also leads to the vulnerable groups of society getting infected by healthcare personnel who should actually be protecting them [17]. There are no uniform diagnostic tools to diagnose SARS-CoV-2 in studies conducted on HCWs. Some have used reverse transcriptase-polymerase chain reaction (RT-PCR) on nasopharyngeal/oropharyngeal swab [22] or IgG/IgM/IgA [8,11] alone while others have employed a combination of the two [23].

The overall seroprevalence of SARS-CoV-2 antibodies among HCWs of the two hospitals in our study was 30.8%. A small community survey in two districts of Karachi, Pakistan revealed that the seroprevalence of SARS-CoV-2 antibodies was 0.2% (95% CI: 0-0.7) in low-transmission areas and 0.4% (95% CI: 0-1.3) in high-transmission areas in the early phase, and 8.7% (95% CI: 5.1-13.1) in low-transmission areas and 15.1% (95% CI: 9.4-21.7) in high-transmission areas in the late phase of the pandemic [6]. A nationwide study has revealed a SARS-CoV-2 seroprevalence of 11% in the general population of Pakistan [7]. The seroprevalence among HCWs in this study is 2-2.8 times higher than the general population of Pakistan. The seroprevalence of SARS-CoV-2 among HCWs is high all over the world. It was reported as 1.07%, 1.6%, 2.7%, 5.1%, 5.3%, 9.3%, and 17.1% from Greece, Germany, Turkey, Italy, US, Spain, and China, respectively [11-15,24,25]. HCWs in our study had a higher seroprevalence of SARS-CoV-2 antibodies than previously reported. Pakistan is a developing country. One-third of the Pakistani population lives below the poverty line (USD 1) and its gross domestic product (GDP) value accounts for only 0.23 percent of the world economy [26]. Pakistan spends only 0.5% of its GDP on health [27]. The higher seroprevalence among our HCWs may be attributed to the insufficient quantity and quality of PPE, poor training regarding standard operating procedures (SOPs), improper donning/doffing, and a higher burden of infection as compared to the developed world. In contrast to the industrialized countries, the majority of patients in Pakistan are brought to the hospital via their personal transportation escorted by their family members/relatives/friends rather than ambulance services. Instead of being looked after entirely by the hospital staff, several relatives often stay within the hospital premises, as part of the culture, as long their patient is staying in the hospital [28]. Because of close contact with their patient (who is suffering from COVID-19) during transportation and inpatient stay, many of the attendants tend to be asymptomatic/pre-symptomatic patients themselves. The HCWs are likely to get infected with SARS-CoV-2 from such asymptomatic/pre-symptomatic attendants and colleagues [29].

As reported during the SARS-CoV-1 epidemic, HCWs working in SARS medical wards had higher seroprevalence than those working in general medical wards [18]. Moreover, it is only common-sensical to assume that HCWs working in COVID-19 receiving facilities will be at a higher risk to contract COVID-19. Surprisingly, the frequency of positive SARS-CoV-2 antibodies for the non-COVID-19 receiving hospital was 32.7% as compared to 28.2% in COVID-19 receiving hospital (p=0.129). These findings are consistent with other studies [11,14,29]. The reasons for the low frequency of seropositivity in the COVID-19 receiving hospital may be due to the staff being more cautious for being in a COVID-19 receiving facility, relatively better provision of PPE, frequent training, and adherence to SOPs for donning/doffing. The provision of standard-quality PPEs, regular training, and taking standard precautions decrease the chances of infection in HCWs. It has been reported during the second phase of the epidemic in China that among the 42,600 HCWs who were deployed in Hubei province to serve the COVID-19 cases, none were infected with SARS-CoV-2 [9]. In contrast, it has been reported from Spain that seroprevalence of SARS-CoV-2 was less frequent (25.8%) in low-risk areas as compared to medium- (33.8%) and high-risk areas (33.1%) (p=0.007) [19]. The findings of the Spanish study could be attributed to the fact that it was conducted only in COVID-19 receiving hospitals with no comparison made with a non-COVID-19 receiving hospital.

Among HCWs who had direct contact with COVID-19 patients, seroprevalence was significantly higher (33.1%) in non-COVID-19 receiving hospital as compared to 23.8% in COVID-19 receiving hospital (p=0.034). In China, the majority of the infected HCWs (89.26%) worked in general hospitals (without in-patient COVID-19 facilities), followed by specialized hospitals (with in-patient COVID-19 facilities) (5.70%) [9]. A study from Germany has indicated that individuals in the high-risk group were protected more as compared to the intermediate-risk group (1.2% vs. 5.4%), with an odds ratio of 0.22 (95% CI: 0.04-1.35); however, the difference was not statistically significant (p=0.13) [11]. In our study, the findings may be attributed to the fact that HCWs of the non-COVID-19 receiving hospital might not have all the required protective gear, and they may have adopted a less careful attitude thinking that they were dealing with non-COVID-19 patients. Non-COVID-19 receiving healthcare facilities were not following and regularly updating their protocols for handling COVID-19 patients. Moreover, there is a major stigma attached to COVID-19 diagnosis in Pakistan. The patients and their attendants have been trying to avoid visiting government hospitals who are admitting COVID-19 patients and reporting patient data to the department of health and national COVID-19 dashboard. The public health section of the health department would vigorously screen all the contacts of the index case, subject the entire family to quarantine under police custody, isolate all seropositive cases, and, in cases of death due to COVID-19, the family members would not be allowed to perform any of the cultural and religious rituals of burial. Therefore, many patients would visit non-COVID-19 receiving institutions, and the patients/attendants would often hide important epidemiological risk factors and sometimes even clinical symptoms to avoid the diagnosis of COVID-19.

The overall seroprevalence in both hospitals ‘A’ and ‘B’ was highest for administrative staff (44%), followed by nursing staff (39.8%), residents (19.8%), and consultants (17.8%) (p=0.001). A higher infection rate of SARS-CoV-2 among nurses than doctors has been reported from Wuhan, China by Zheng et al. The case infection rate (CIR) of nurses was markedly higher than that of physicians (p=0.001) [9]. In Italy, the frequency of COVID-19 was high among physicians (10.6%, 95% CI: 8.3-13.4) as compared to that of clerical workers and technicians (2.9%, 95% CI: 0·8-7.3) [30]. There are contradictory reports about the risk of contracting COVID-19 concerning staff categories [17]. The high infection rate among nursing staff compared to doctors (both consultants and residents) may be due to nurses' longer and closer physical contact with the patients, improper use of PPE, and non-adherence to proper SOPs. The high seroprevalence among the administrative staff may be due to their being less cautious, resulting in inadequate protection, and due to overexposure to the relatives of COVID-19 patients who would visit them for the provision of free medicines, allotment of single rooms, and other petty matters, which is very common in Pakistan. Additionally, the administrative offices were openly accessed by hospital staff and relatives alike.

There was no gender- or age group-wise difference in the seroprevalence among HCWs of both hospitals (p: >0.05). Similar findings have been reported by other researchers [9,14].

The subgroup analysis in COVID-19 receiving hospital revealed that the seroprevalence was 38.6% in HCWs having no close/direct contact with COVID-19 patients as compared to 23.8% among those having close/direct contact (p=0.002), with an odds ratio of 1.75 (95% CI: 1.11-2.77). Similar findings were reported by a study conducted in Greece [14].

## Conclusions

Based on our findings, seropositivity to COVID-19 does not depend on the COVID-19 receiving status of the hospital. Seroprevalence was significantly higher in HCWs who had direct contact with patients in the non-COVID-19 receiving hospital. Overall, seroprevalence was highest for administrative staff, followed by nursing staff, residents, and consultants. The higher seroprevalence found among the HCWs highlights the importance of the provision of adequate PPE to HCWs, regular training, and insistence on adherence to SOPs for donning and doffing irrespective of the COVID-19 receiving status of the healthcare facilities.
